# Enhanced Anti-Atherogenic Effects of Epicatechin and Hydroxytyrosol in THP-1 Macrophages: An Integrated In Silico and In Vitro Study

**DOI:** 10.3390/ijms27104235

**Published:** 2026-05-10

**Authors:** Noor Omar Bashanfar, Etimad Huwait, Maryam A. Al-Ghamdi, Zeenat Mirza

**Affiliations:** 1 Department of Biochemistry, Faculty of Sciences, King Abdulaziz University, Jeddah 21589, Saudi Arabia; noor-omar96@hotmail.com (N.O.B.); ehuwait@kau.edu.sa (E.H.); maaalghamdi3@kau.edu.sa (M.A.A.-G.); 2 Cell Culture Lab, Experimental Biochemistry Unit, King Fahd Medical Research Centre, King Abdulaziz University, Jeddah 21589, Saudi Arabia; 3 EcoHealth Unit, King Fahd Medical Research Center, King Abdulaziz University, Jeddah 21589, Saudi Arabia; 4 Department of Medical Laboratory Sciences, Faculty of Applied Medical Sciences, King Abdulaziz University, Jeddah 21589, Saudi Arabia

**Keywords:** epicatechin, hydroxytyrosol, human THP-1 macrophage, atherosclerosis, MCP-1, ROS, molecular docking

## Abstract

Atherosclerosis is caused by inflammatory processes that alter the permeability of arterial wall cells and leucocyte recruitment, leading to oxidation of low-density lipoproteins in the artery. The use of dietary polyphenols as antioxidants seems promising. Herein, molecular docking-based screening was initially used to predict the interactions of epicatechin and hydroxytyrosol on multiple cytokines that can trigger atherosclerosis development. Computational results show that epicatechin and hydroxytyrosol interact with the cytokines, namely, intercellular adhesion molecule-1, vascular cell adhesion molecule-1, monocyte chemoattractant protein 1 (MCP-1), granulocyte–macrophage colony-stimulating factor, leukocyte differentiation antigen CD36, and oxidized low-density lipoprotein receptor-1. Cytotoxicity of both the bioactive compounds to human monocytic THP-1 macrophages was evaluated by lactate dehydrogenase and crystal violet assays. ROS activity evaluation was done for the phytocompounds followed by monocyte migration assay for MCP-1. The expression levels of selected biomarkers were further assessed by quantitative polymerase chain reaction. Inhibition of these atherosclerotic biomarkers may limit the atherogenic effect. Notably, these two polyphenols at a concentration of 0–125 µg/mL for 24 h showed no cytotoxicity on THP-1 macrophages and exhibited decreased ROS production and MCP-1 levels. The genes implicated in the early stages of inflammation are potential therapeutic targets to effectively reduce atherogenesis and prevent CVD. The interaction between the selected cytokines and the two natural compounds indicates their potential ability to inhibit the inflammation in vitro and exhibit anti-atherogenic effects. Hence, epicatechin and hydroxytyrosol possess significant anti-atherosclerotic effects and, in combination, could contribute positively to the treatment of atherosclerosis.

## 1. Introduction

Globally, cardiovascular disease (CVD) continues to represent a significant health and economic burden to society, which is no longer limited to industrialized countries due to the widespread and increasing prevalence of risk factors such as obesity, hyperlipidemia, and diabetes [1]. Atherosclerosis, the main cause of CVD, is a chronic, low-grade inflammatory disease of the vascular system that develops over decades [2]. In inflammatory sites, the endothelium is stimulated to express many adhesion molecules such as intercellular adhesion molecule-1 (ICAM-1), vascular cell adhesion molecule-1 (VCAM-1), and selectins that mediate monocyte binding. Upon monocyte binding, these adhesion molecules activate endothelial cell signaling through nicotinamide adenine dinucleotide phosphate (NADPH) oxidase-triggered reactive oxygen species (ROS) formation and alter endothelial cell shape for the opening of passageways through which monocytes and low-density lipoprotein (LDL) can migrate [3]. NADPH oxidase produces a superoxide anion from O_2_, which is converted to hydrogen peroxide (H_2_O_2_) [4]. The increase in H_2_O_2_ leads to failure of the antioxidant defense system by triggering production of matrix metalloproteinases and the protein kinase Cα signal [5,6]. This imbalance leads to increased oxidative stress, endothelial dysfunction, and monocyte migration, which are a major determinant of the initial stage of atherogenesis.

The high LDL cholesterol and excessive ROS that diffuse from the intima of the blood vessel oxidize to ox-LDL [7,8]. The ox-LDL, having altered density, chemical properties, and size, obstructs the blood flow in the vessel wall [8]. Ox-LDL impacts atheroma development by suppressing the normal production of endothelial nitric oxide synthase and promoting ROS generation from smooth muscle cells and macrophages [7]. This induces the expression of adhesion molecules on endothelial cells, synthesis of collagen, migration of smooth muscle cells, and platelet activation through internalization and uptake of modified oxidized LDL (ox-LDL) into macrophages by various scavenger receptors (SRs) such as SR-A types I and II, oxidized low-density lipoprotein receptor-1 (LOX-1), and leukocyte differentiation antigen-36 (CD36) [8]. The LOX-1 scavenger receptor is the most potent activator of nuclear factor kappa-light chain enhancer of activated B cells (NF-κB) that triggers increased expression of adhesion molecules (cytokines) like VCAM-1 and monocyte chemoattractant protein-1 (MCP-1) [4,9]. Growth factors, for instance, granulocyte–macrophage colony-stimulating factor (GM-CSF), stimulate the expression of various SRs (CD36 and LOX-1) and differentiation of monocytes into macrophages that absorb altered lipids (ox-LDL), leading to the creation of foam cells, a key initial feature of atherosclerotic plaques. The cytokines induce elevated LOX-1 expression in endothelial cells, creating a continuous flow of signal between ox-LDL, LOX-1, and NF-κB [10]. A recent study indicates that the absorption of ox-LDL in macrophages from mice lacking SR-AI and SR-AIII is decreased by around 60% [11]. In addition, human macrophages lacking CD36 show a 40% decrease in their ability to attach and absorb ox-LDL [12]. Knock-out of CD36 and LOX-1 causes ischemia damage, making the scavenger receptor a promising target for the therapy of atherosclerosis and related diseases [11,13].

Flavonoids are polyphenolic compounds that are found naturally in fruit and vegetables and have a positive effect on blood vessels [14,15]. Known phenolic compounds such as anthocyanins and catechins promote vasodilation and bestow antioxidant effects [16,17]. Although there is a wealth of evidence for the cardioprotective effects of foods rich in flavanols, the effects of individual flavanols in relation to atherosclerosis remain less explored. Nevertheless, there is a lack of studies (both in animal models and humans) investigating the effects and underlying mechanisms of combined flavanols in relation to atherosclerosis. Epicatechin (Epi) is a polyphenol characterized by a basal structure with two benzene rings. Epi metabolites significantly promote vascular health through epigenetic reprogramming of endothelial immune cell signaling and reverse low-level systemic inflammation [18]. Hydroxytyrosol (HT), a non-flavonoid polyphenolic compound derived from oleuropein, is an efficient bioactive phenolic candidate with antioxidant, anti-inflammatory, and anti-atherogenic effects due to its protective action against inflammation and oxidative stress [19]. HT reportedly reduces lipid peroxidation of LDL cholesterol, one of the main causes of atherosclerosis [20].

Molecular docking is a method used for drug discovery that is based on the estimated binding free energy and inhibition constant (Ki) between the compound of interest and the target proteins [21]. The 3D structure of proteins was used for rational screening to evaluate molecules of therapeutic interest based on structural and electrostatic ligand complementarity. The interaction study of the selected polyphenolic compounds with the atherosclerotic biomarkers was performed to get an idea of the inhibition. This virtual screening approach fastens and reduces the experimental cost [22,23]. Despite the extensive literature documenting their anti-inflammatory properties, the precise molecular targets—particularly specific receptors and cytokines involved in the early stages of atherosclerosis—remain insufficiently defined. Addressing this gap, the present study investigates the mechanistic basis of their action. Hence, the therapeutic use of dietary supplements to reduce complications associated with atherosclerosis and cardiovascular diseases needed a spotlight, and an attempt has been made herein.

## 2. Results

This computational study, coupled with biochemical assays, aimed to understand the mechanism of atherosclerosis in its early stages and the interaction of natural compounds as inhibitors of major inflammatory proteins. This approach seems promising to reduce the uptake of ox-LDL in macrophages and reduce lipid deposition in arteries. Polyphenols (Epi and HT and combination) offer antioxidant, anti-atherogenic, and anti-inflammatory properties. Inflammatory protein biomarkers, namely, ICAM-1, VCAM-1, MCP-1, GM-CSF, and scavenger receptors (CD36 and LOX-1), activate monocyte migration via production of adhesion molecules, which makes it easier for inflammatory proteins (cytokines) to adhere to and circulate monocytes in response to chemotactic signals [9]. Further validation was done by testing the viability and proliferation of THP-1 macrophages, ROS activity, and monocyte migration.

### 2.1. Prediction of Protein–Protein Interaction

**Figure 1:**
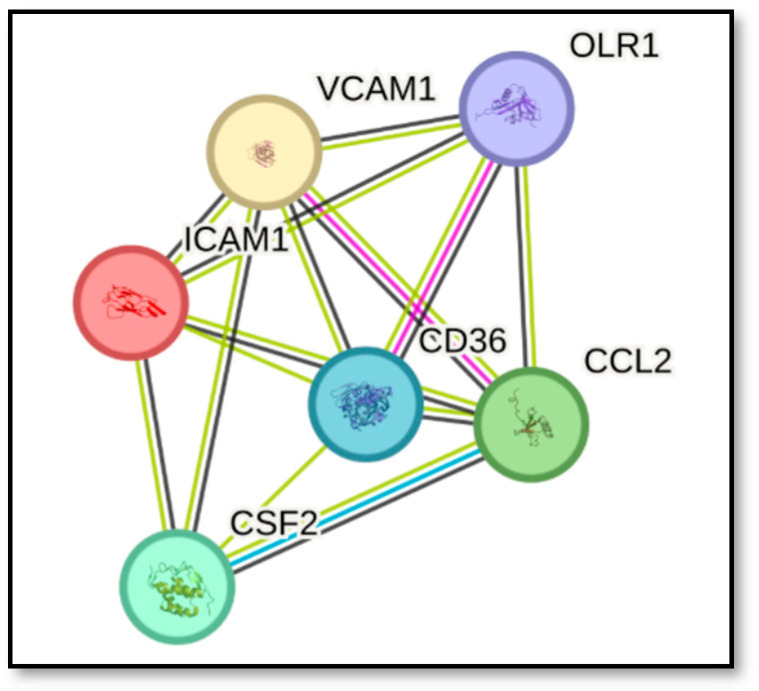
The protein–protein interaction graph was generated using STRING v 12.0 [24] following standard visualization parameters. Here nodes represent proteins and edges represent interaction evidence (color-coded by type) and confidence scores (line thickness). Colors of the edges indicate known interactions from curated databases (light blue), experimentally determined interactions (magenta), gene neighborhood (green) and text-mining sources (black).

The generated protein–protein interaction (PPI) network using STRING v 12.0 [24] shows the interrelationship between the six inflammatory atherosclerosis biomarkers. It demonstrated significant functional connectivity among the selected proteins, with a statistically significant enrichment *p*-value, indicating that the observed interactions are biologically meaningful rather than random (Figure 1). This association indicates that the proteins are jointly contributing to a shared function, but this does not necessarily mean they are physically binding to each other. Each connection has support of text mining and co-expression. The specific interactions between VCAM-1 and MCP-1 (CCL2) and CD36 and LOX-1 (OLR1) have proofs of experimental determination. The interactions between GM-CSF and MCP-1 have been established from curated databases.

### 2.2. Chemoinformatic Prediction

**Figure 2:**
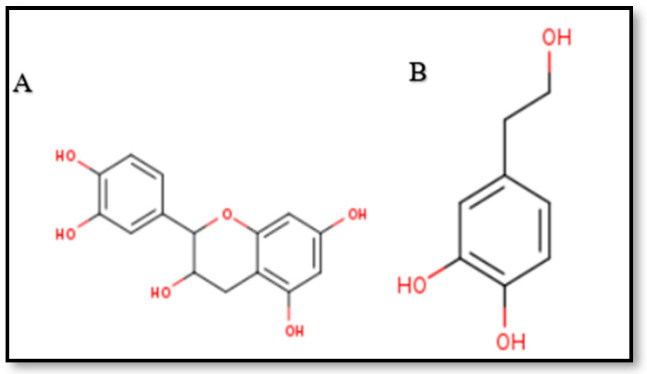
2D chemical structure of (**A**) epicatechin and (**B**) hydroxytyrosol.

Physicochemical analysis was done using the web-based SwissADME tool [25] for Epi and HT monomer, as described in Section 4.1. The in silico predictions of physicochemical properties were generated for Epi and HT, which illustrate that both are hydrophilic (MLogP = 0.24 and 0.60, respectively). The 2D chemical structures of the two ligands (Epi and HT) are shown in Figure 2. Epi has a greater number of hydrogen bond (H-bond) acceptors and donors and a lesser number of rotatable bonds as compared to HT (Table 1).

### 2.3. Molecular Docking and Binding Site Prediction

Molecular docking studies predicted the interaction between the selected biomarker proteins and the polyphenols. The binding affinity estimation of Epi and HT with target proteins was made based on the root mean square deviation being less than 2 Å. A lower binding energy indicates a better affinity for the protein–ligand complex. Epi and HT showed higher binding affinity and the lowest inhibition constant to the six target inflammatory proteins, as summarized in Table 2.

**Figure 3:**
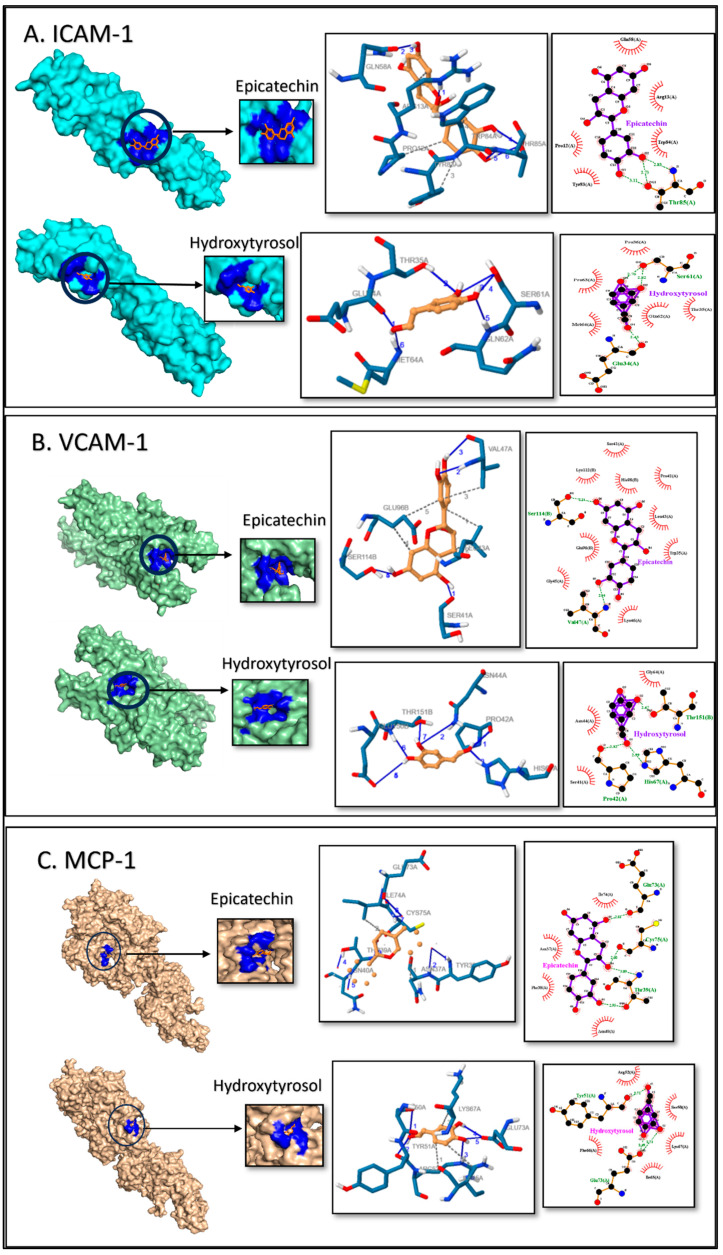
Analysis of docking by protein-bound ligands’ 3D structure, protein–ligand interaction profiler (PLIP), and LigPlot+ exhibiting 2D ligand-binding interactions of (**A**) ICAM-1, (**B**) VCAM-1, and (**C**) MCP-1 bound with Epi and HT and magnified ligand-binding sites, respectively. Residues are labeled with their amino acid name and sequence number. In the PLIP figures, ligand is in orange color and the protein residues are colored blue and H-bonds are represented by blue lines and black dashed lines represent hydrophobic interactions. In the LigPlot+ H-bonds are shown by green dashed lines and brick red represents hydrophobic interactions. Protein residue chain identifiers are represented by (**A**,**B**).

The binding of two compounds, Epi and HT, to ICAM-1 showed potential interactions and comparable binding energies (−4.69 and −4.76 kcal/mol, respectively). Polar H-bond interactions existed between Epi in Thr85N-O2 (2.83 Å), Oγ1-O2 (2.73 Å), and Oγ1-O3 (3.11 Å) (Figure 3A). Several hydrophobic interactions were present, namely with Pro12, Arg13, Gln58, Tyr83, and Trp84. HT binding was slightly stronger than Epi, indicating a potentially more stable interaction due to polar H-bond interactions amid Glu34O-O1 (2.43 Å), Ser61Oγ-O2 (2.82 Å), and Oγ-O3 (2.76 Å) as well as several hydrophobic interactions in Thr35, Pro36, Gln62, Pro63, and Met64 (Figure 3A).

The binding energy of VCAM-1 with Epi was −3.64 kcal/mol. The polar interactions via H-bonds noticed with Epi were Val47N-O2 (2.94 Å) and Ser411OG-O6 (3.21 Å). Also, several hydrophobic interactions were noted with Trp35, Ser41, Pro42, Leu43, Gly45, Lys46, Glu96, His98, and Lys112 (Figure 3B). HT has a significantly stronger binding affinity to VCAM-1 (−5.21 kcal/mol). The polar interaction was formed by H-bonds with Pro42O-O1 (2.85 Å), His67NE2-O1 (2.67 Å), and Thr151OG1-O2 (2.67 Å), and hydrophobic interactions were also observed with Ser41, Asn44, and Gly64 (Figure 3B). Both compounds show significant hydrophobic interactions with various residues of VCAM-1, indicating more stability and specificity in the binding.

The MCP-1 showed an optimal binding to Epi and HT (−5.65 and −4.83 kcal/mol, respectively). Epi has polar interaction via H-bonds with Thr36Nam-O4 (3.68 Å), Asn37Nam-O3 (3.83 Å), Thr39Nam-O4 (3.09 Å), Thr39O3-O3 (2.95 Å), Asn40Nam-O3 (3.23 Å), Glu173O-O2 (2.781 Å), and Cys75Nam-O3 (3.55 Å). Also, stronger hydrophobic interactions were seen with Ile74; Asn37 might contribute to a more stable interaction with MCP-1. Similarly, HT forms H-bonds with Ser50O-O3 (3.80 Å), Tyr51O-O3 (2.71 Å), Arg52Ng^+^-O3 (3.77 Å), Glu73O-O3 (2.71 Å), and Glu73O-O3 (2.49 Å), also indicating a more stable interaction with MCP-1. Rest hydrophobic interactions were seen with Lys67, Arg52, and Ile65 (Figure 3C).

**Figure 4:**
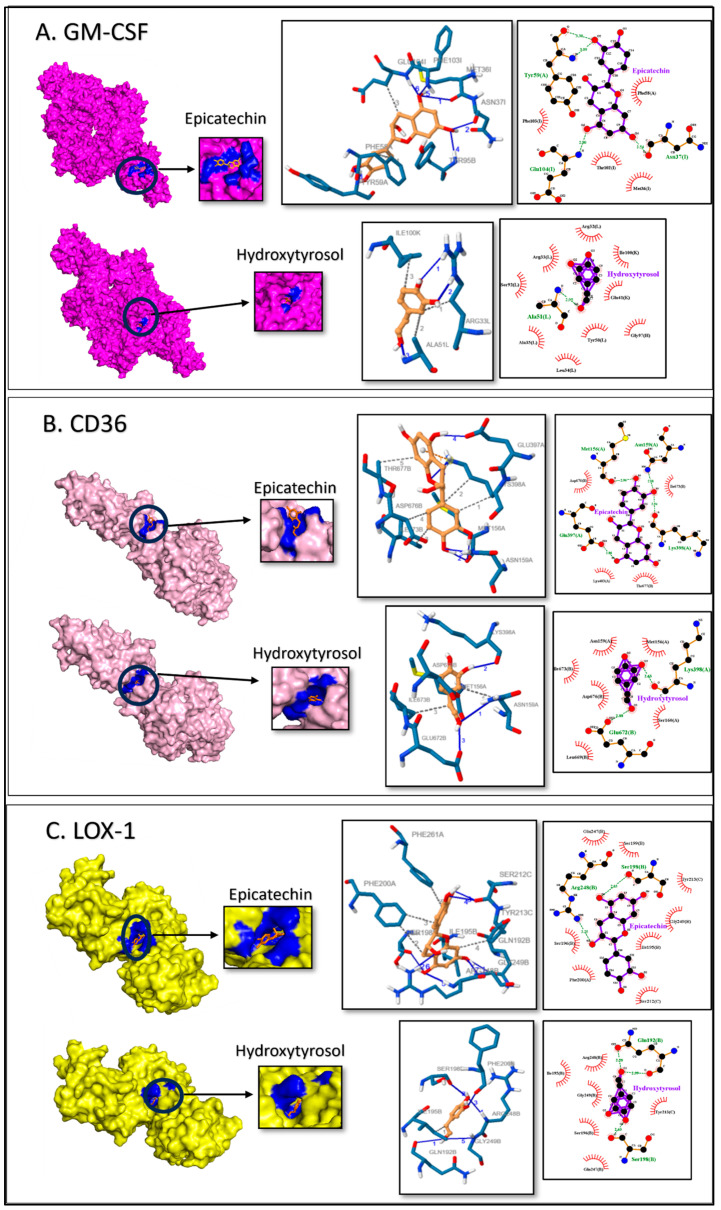
Analysis of docking by protein-bound ligands’ 3D structure, protein–ligand interaction profiler (PLIP), and LigPlot+ exhibiting 2D ligand-binding interactions of (**A**) GM-CSF, (**B**) CD36, and (**C**) LOX-1 bound with Epi and HT and magnified ligand-binding sites, respectively. Residues are labeled with their amino acid name and sequence number. In the PLIP figures, ligand is in orange color and the protein residues are colored blue and H-bonds are represented by blue lines and black dashed lines represent hydrophobic interactions. In the LigPlot+ H-bonds are shown by green dashed lines and brick red represents hydrophobic interactions. Protein residue chain identifiers are represented by (**A**,**B**).

The binding free energy of GM-CSF to Epi and HT was almost similar (−4.39 and −4.71 kcal/mol, respectively). The polar H-bond interactions of Epi with GM-CSF involved multiple polar contacts spread across different regions formed between Asn37O-O6 (2.54 Å), Tyr59N-O2 (3.09 Å), and Glu104N-O5 (2.80 Å). Also, several hydrophobic interactions were present, namely with Met36, Phe85, Thr102, and Phe103, while the H-bonds in HT formed only with AlaN-O1 (2.95 Å), suggesting a more localized interaction with GM-CSF. The rest of the hydrophobic interactions were noticed with Arg32, Arg33, Leu34, Ala53, Glu41, Tyr50, Ser92, Gly97, and Ile100, indicating that the interactions have a stronger binding energy (Figure 4A).

Epi shows a good binding affinity to CD36 (−5.22 kcal/mol) and has a broader range of interaction. The polar interactions mainly seen were the H-bonds formed in Asn159ND2-O2 (2.88 Å), Met156O-O3 (2.96 Å), Glu397OE1-O5 (2.86 Å), and Lys398O-O2 (2.94 Å). Several hydrophobic interactions were present in Lys403, Ile673, Asp676, and Thr677, which may have a potent effect on CD36. Similarly, the binding energy of HT to CD36 was −5.04 kcal/mol via the H-bonds formed with Lys398O-O2 (2.63 Å) and Glu672OE2-O1 (2.88 Å). Other hydrophobic interactions were noticed with Met156, Asn159, Ser160, Leu669, Ile673, and Asp676 (Figure 4B); the significant binding interaction might be useful for targeted modulation of CD36.

Epi and HT bind with high affinity to LOX-1 (−6.93 kcal/mol and −5.84 kcal/mol, respectively). The polar interactions mainly seen were the H-bonds formed with Epi and Ser198O-O5 (2.65 Å) and Arg248NH1-O4 (3.25 Å), which resulted in a more stable interaction with LOX-1. Several hydrophobic interactions were present, namely with Ile195, Ser196, Ser199, Phe200, Ser212, Tyr213, Glu247, and Gly249. Similarly, HT has polar interactions via the H-bonds formed with Glu192OE1-O1 (2.58 Å) and Glu192 O-O1 (2.99 Å) and Ser198O-O3 (2.63 Å). The remaining hydrophobic interactions were noticed with Ile195, Ser196, Tyr213, Gln247, Arg48, and Gly249 (Figure 4C) and contributed to a strong but more localized interaction.

### 2.4. Effect of Epi and/or HT on Cell Viability and Proliferation of Human THP-1 Macrophages

**Figure 5:**
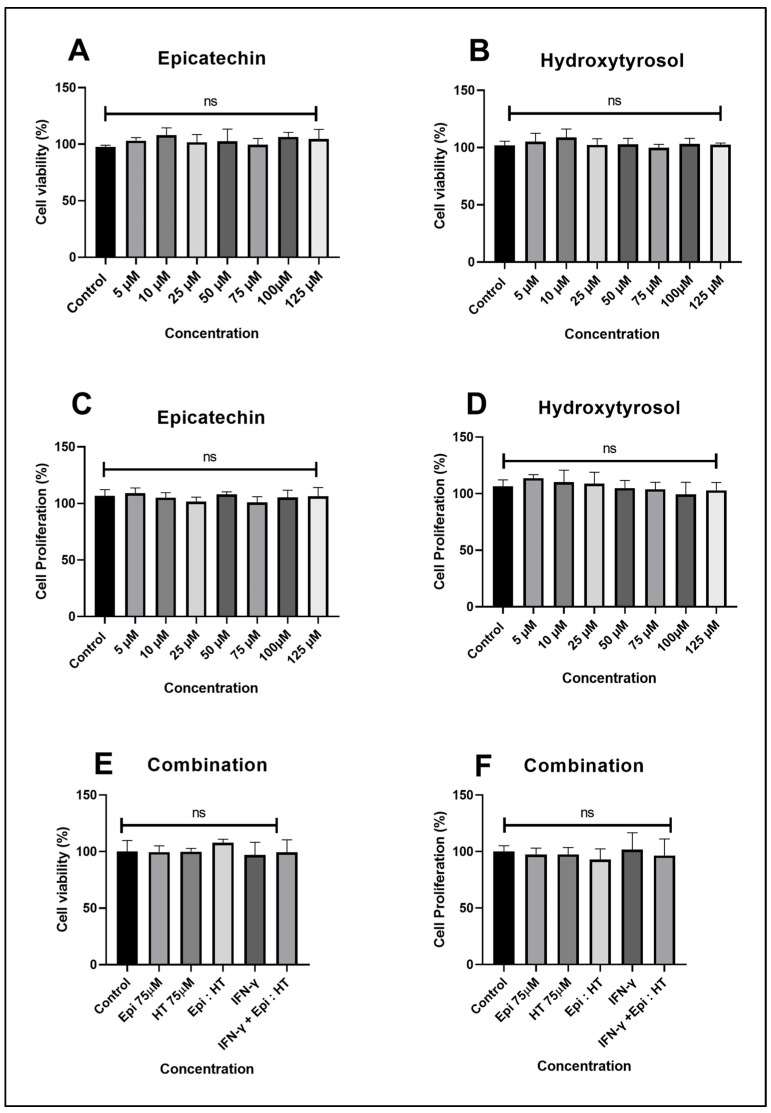
Biological activity of Epi and HT on macrophages 24 h later. Percentage of cell viability with Epi (**A**) and HT (**B**). Percentage of cell proliferation with Epi (**C**) and HT (**D**). Percentage of cell viability of combination of Epi and HT (**E**). Percentage of cell proliferation of combination of Epi and HT (**F**). All the graphs present data as the mean ± SEM from three independent experiments. Statistical analysis was performed using GraphPad Prism analysis; the *p*-value was ns (indicating not significant at *p* > 0.05). Abbreviations: Epi—epicatechin; HT—hydroxytyrosol.

Lactate dehydrogenase assay and crystal violet stain were used to measure the effect of compounds (Epi and HT) on the cell viability and proliferation in human THP-1 macrophages, a model of atherosclerosis. This was carried out to ensure that there was no significant cytotoxic effect on cells. A range from 5 to 125 µg/mL was used to identify optimal concentration for dose–response experiments that can subsequently be used for further studies in this cell culture model. No significant effects on cell viability were observed following treatment of the cells with Epi (Figure 5A), HT (Figure 5B), and the combination (Figure 5E) for 24 h when compared to the control in THP-1 macrophages. Also, no significant changes were seen in cell proliferation on THP-1 macrophages after 24 h of treatment with Epi and HT and their combination when compared to the control (Figure 5C,D,F). Therefore, 75 µg/mL of Epi and 75 µg/mL of HT were chosen for further investigation.

### 2.5. Epi and/or HT Significantly Inhibits THP-1 Monocyte Migration to MCP-1

**Figure 6:**
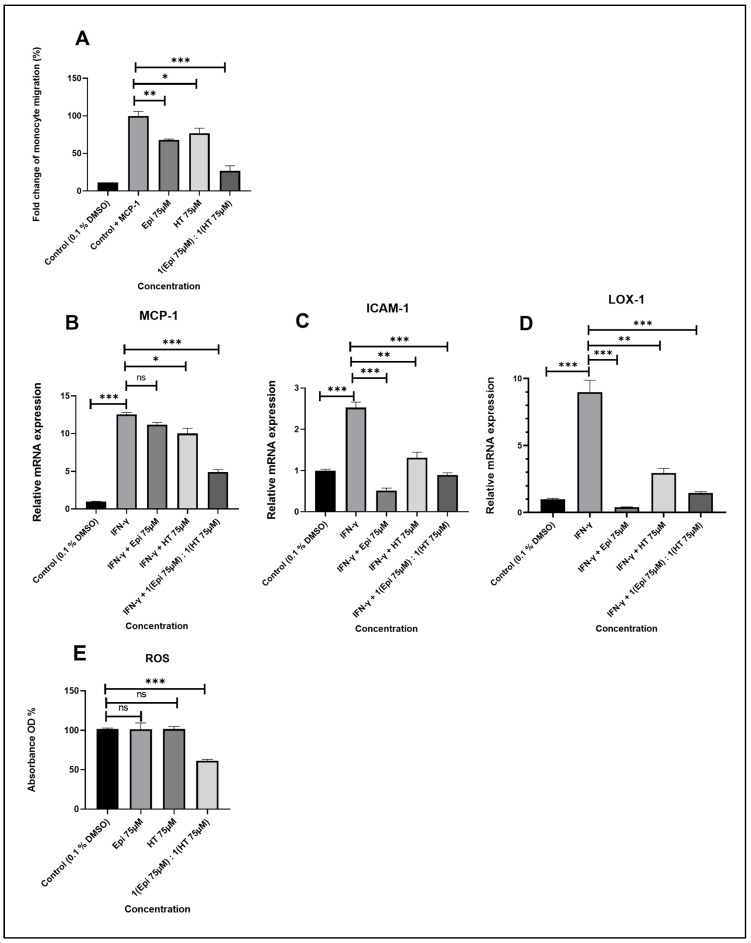
(**A**) Monocyte migration is induced via MCP-1. THP-1 monocytes were incubated with vehicle control alone or MCP-1 (20 ng/mL) with 75 µg/mL of Epi and/or HT alone or in combination for 3 h. mRNA expression levels of MCP-1 (**B**), ICAM-1 (**C**), and LOX-1 (**D**) were evaluated in THP-1 macrophages. The cells were induced with IFN-γ for 3 h, then treated with 75 µg/mL of Epi and/or HT alone or in combination. (**E**) ROS production was assessed in PMA-differentiated THP-1 human macrophages treated either with control or in the presence of Epi or HT alone or in combination. All graphs present data as the mean ± SEM from three independent experiments. Statistical significance was determined by one-way ANOVA followed by Tukey’s post hoc test using GraphPad Prism analysis; the *p*-value was significant when * *p* ≤ 0.01, ** *p* ≤ 0.001, *** *p* ≤ 0.0001; ns means not significant. Abbreviations: Epi—epicatechin; HT—hydroxytyrosol; IFN—γ-interferon gamma; MCP-1—monocyte chemoattractant protein-1; ICAM—1-intercellular adhesion molecule-1; LOX-1—oxidized low-density lipoprotein receptor-1.

Cell migration during atherosclerosis is a key mechanism in lesion formation [26,27]. MCP-1, a key chemokine in atherosclerosis, is reportedly known to stimulate cellular migration [28,29]. As shown in Figure 6A, THP-1 monocyte migration was significantly increased by 95% in the presence of 20 ng/mL MCP-1 for 3 h when compared to vehicle alone (no MCP-1). Treatment of cells with Epi and HT (75 µg/mL concentration) alone inhibits MCP-1-driven migration, showing a reduction by around 40–50% respectively (*p* ≤ 0.01, *p* ≤ 0.001). However, the treatment of cells with Epi and HT in combination inhibits MCP-1-driven migration by around 80% (*p* ≤ 0.0001).

### 2.6. Epi and/or HT Modulates the Expression of Inflammatory Markers

MCP-1, ICAM-1, and LOX-1 are critical inflammatory genes involved in endothelium dysfunction. The transcriptomic expression of these genes in THP-1 macrophages following treatment with Epi and/or HT alone, either individually or in combination after stimulation with IFN-γ, is presented in Figure 6.

Quantitative real-time PCR analysis demonstrated that stimulation with IFN-γ significantly increased the expression levels of the inflammatory genes MCP-1, ICAM-1, and LOX-1 compared with the control group. MCP-1 expression was markedly upregulated to approximately 12.56-fold, while ICAM-1 and LOX-1 were increased to 2.52-fold and 8.99-fold, respectively.

Treatment with epicatechin (75 µM) reduced the expression levels of MCP-1, ICAM-1, and LOX-1 to 11.20-fold, 0.52-fold, and 0.40-fold, respectively. Similarly, treatment with hydroxytyrosol (75 µM) decreased the expression of MCP-1, ICAM-1, and LOX-1 to 10.00-fold, 1.31-fold, and 2.95-fold, respectively. Notably, the combined treatment of epicatechin and hydroxytyrosol (1:1) showed a stronger inhibitory effect, reducing MCP-1, ICAM-1, and LOX-1 expression to 4.93-fold, 0.90-fold, and 1.47-fold, respectively, compared with the IFN-γ-treated group.

### 2.7. Effect of Epi and/or HT on ROS Production

The maintenance of cellular redox status is necessary for homeostasis in the vascular system. The effect of Epi and HT alone and in combination with ROS production was measured in THP-1 macrophages. Treatment with a concentration of 75 µg/mL of Epi and 75 µg/mL of HT alone had no significant effect on ROS production when compared to the control, while it showed a significantly reduced effect by 40% (*p* ≤ 0.0001) with 75 µg/mL of Epi and 75 µg/mL of HT in combination when compared to the control (Figure 6E).

## 3. Discussion

**Figure 7:**
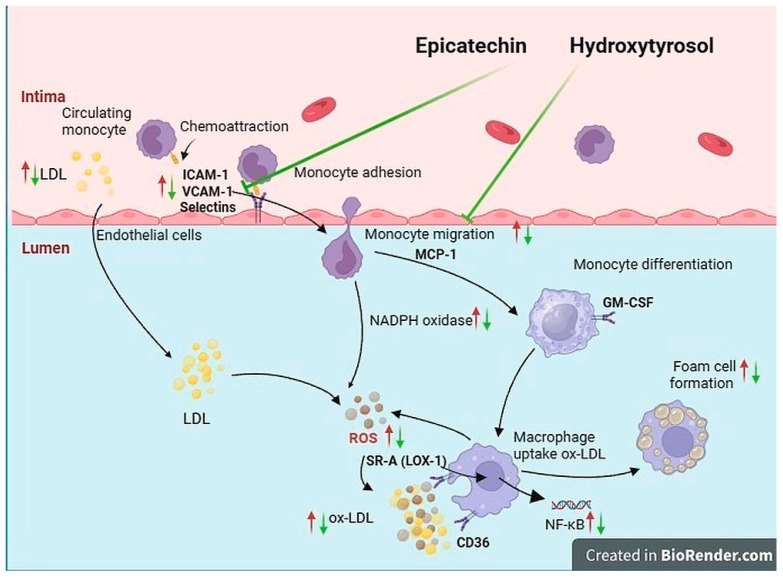
Mechanism of atherosclerosis. The oxidation of LDL to ox-LDL leads to the activation of adhesion molecules (ICAM-1 and VCAM-1), which brings monocytes to the sub-endothelial area due to chemotactic signals (MCP-1). GM-CSF induces monocyte differentiation to macrophages, causing the expression of scavenger receptors (CD36 and LOX-1) and uptake of ox-LDL, followed by foam cell formation. The red arrow exhibits the overexpression of the inflammatory biomarkers leading to atherosclerosis development. Epi and HT potentially inhibit the mechanism of atherosclerosis (green arrow). (Illustration made using BioRender; adapted from [9]).

Modulation of inflammatory processes may prevent the onset and progression of early-stage atherosclerosis. Phenolic compounds demonstrate anti-inflammatory properties by inhibiting the expression of pro-inflammatory mediators, suppressing immune cell activation, and reducing oxidative stress [30]. MCP-1 is a key chemokine involved in the recruitment of monocytes to the vascular endothelium, while ICAM-1 facilitates leukocyte adhesion and transmigration across the endothelial layer. In addition, LOX-1 plays an essential role in the uptake of oxidized low-density lipoprotein (ox-LDL), contributing to foam cell formation and the progression of atherosclerosis [9] (Figure 7).

Computational studies were conducted with all the six selected inflammatory biomarker proteins. Network analysis revealed strong functional associations between ICAM-1 and VCAM-1, reflecting their coordinated regulation during endothelial activation. The functional linkage between MCP-1 and GM-CSF, suggesting cytokine-mediated amplification of monocyte recruitment and macrophage activation. Also, close interaction clustering occurred between LOX-1 and CD36, highlighting their shared role in oxidized LDL recognition and foam cell formation.

Collectively, the PPI network supports the concept that these molecules form an integrated inflammatory and lipid-driven signaling axis, contributing to endothelial dysfunction, monocyte recruitment, macrophage activation, and foam cell formation during atherogenesis. Importantly, the interactions observed were predominantly functional associations rather than direct physical binding events, reflecting coordinated transcriptional regulation within inflammatory signaling cascades.

The interactions formed by hydrogen and hydrophobic bonds with specific amino acid residues play a key role in the stability and specific binding of the complex. Both Epi and HT show comparable binding energy to ICAM-1; polar amino acid residues (Glu34 and Ser61 in Epi and Thr85 in HT) especially exhibit hydrogen bonds and several hydrophobic interactions, indicating important sites for the potential modulation of ICAM-1 activity. as Additionally, in VCAM-1, the strong binding energy, especially the H-bonds with specific amino acids (Thr85), could enhance the potential binding of these two compounds.

MCP-1 has strong binding interactions with Epi that spread across different regions of protein. This might contribute to more stable interactions. While HT forms fewer H-bonds, the interactions are more localized at the binding site. These bindings may reduce the activity of MCP-1 in the migration of monocytes to the intima in arteries. GM-CSF displays high binding energy with H-bond in Epi and a broader range of hydrophobic interaction with HT that could exert a potent effect on GM-CSF activity in differentiation to macrophages. CD36 and LOX-1 are scavenger receptors in macrophages that show strong binding, indicating that they interact both via multiple H-bond and hydrophobic interactions with Epi and HT. These binding interactions indicate how Epi and HT regulate CD36 and LOX-1 activity, which is relevant for developing treatment target oxidative stress and inflammation.

The human leukemia-derived monocytic cell line, THP-1, has been widely used in vitro to study monocyte/macrophage function and the effects of anti-inflammatory drugs [31,32,33]. There is an increased interest in their use as a potential natural alternative to pharmaceutical drugs for the prevention and treatment of inflammation and related diseases with minimal side effects [5]. However, more in-depth studies are required to comprehend their mechanisms of action.

The PMA induction for 24 h differentiates THP-1 into macrophages [31,32,33]. The viability and proliferation of treated THP-1 macrophages were like those of the control cells, which implied that Epi and HT had no cytotoxicity. Our present findings are in accordance with earlier studies wherein Epi and HT alone in the THP-1 cell line were incubated overnight with various concentrations up to 160 μM for Epi [34] and up to 200 μM for HT [35]. No significant differences were noticed in cell viability and proliferation in THP-1 macrophages post-treatment with Epi and HT as compared to the control. This seems consistent with the findings in the previously published literature [34,35].

The decrease in MCP-1 levels leads to a reduction in lipid deposition in arteries [29,36]. Our study indicated that Epi and HT alone and in combination inhibited monocytes recruited via MCP-1. Therefore, Epi and HT might help to prevent chemokine stimulation and the migration of monocytes [36] (Figure 7).

The qPCR results demonstrate that stimulation with IFN-γ markedly increased the expression of the inflammatory genes MCP-1, ICAM-1, and LOX-1 in THP-1 macrophages, confirming the strong pro-inflammatory role of IFN-γ in promoting endothelial dysfunction and atherosclerotic processes, an indicator of successful in vitro arterial modeling [37,38]. The significant upregulation of these genes following IFN-γ stimulation observed in this study is consistent with previous reports demonstrating that inflammatory cytokines activate macrophages and enhance the expression of adhesion molecules and chemokines associated with vascular inflammation [37].

Previous research showed that the potent anti-inflammatory effects of Epi and HT alone inhibited several macrophage processes associated with atherosclerosis: monocytic migration, expression of pro-atherogenic genes, ROS production, uptake of modified LDL, and stimulation of the inflammasome and the inflammatory signaling cascade [34,39]. In addition, HT has been shown to modulate inflammatory responses and protect against oxidative stress-induced vascular damage [40] and stimulate the efflux of cholesterol from foam cells, upregulate the expression of the ABCA1 gene [39], block VCAM-1 signal transduction [3], and lead to the inhibition of NF-κB signaling [34].

Notably, the combined treatment of Epi and HT produced a stronger inhibitory effect on gene expression than either compound alone, indicating a potential enhanced action between the two polyphenols. This combined effect may enhance the suppression of inflammatory pathways involved in macrophage activation and endothelial dysfunction.

In this study, the combined treatment of epicatechin (Epi) and hydroxytyrosol (HT) demonstrated the potential to regulate key biomarker genes involved in the early stages of atherosclerosis by reducing reactive oxygen species (ROS) production and inhibiting in vitro monocyte migration. These effects were associated with the modulation of MCP-1 expression, which may contribute to decreased uptake of modified low-density lipoprotein (LDL), reduced scavenger receptor activity in THP-1 macrophages, and attenuation of the ox-LDL/LOX-1/NF-κB signaling pathway. Furthermore, molecular docking analyses suggested that Epi and HT may interact with relevant binding sites of target proteins, supporting their potential role in modulating pathways implicated in early atherogenesis. Docking results provide preliminary insights into potential interactions and do not confirm functional inhibition without further experimental validation.

The primary aim of the present study was to investigate “transcriptional regulation” of inflammatory and lipid metabolism genes (ICAM-1, VCAM-1, MCP-1/CCL2, CD36, LOX-1) in THP-1 macrophages following exposure to Epi and HT. Multiple recent studies have validated that mRNA changes for our specific targets correlate strongly with protein-level changes in THP-1 and related models [41,42]. RT-qPCR was therefore the most appropriate method to address this specific objective. This study is limited by the absence of protein-level validation (e.g., Western blot or ELISA), which will be addressed in future investigations.

A limitation of the RT-qPCR analysis is the use of a single reference gene (GAPDH) for normalization, rather than the recommended two or more genes according to MIQE guidelines. However, we verified that GAPDH Ct values varied by <0.5 cycles across all experimental groups (THP-1 cells treated with Epi or HT), confirming its stability. Due to exhausted RNA samples, re-analysis with an additional reference gene was not possible. The raw Ct values for GAPDH are provided in Supplementary Table S1 to allow readers to independently assess its stability. Therefore, the gene expression results presented here should be interpreted as supportive rather than definitive quantitative measurements. Future investigations should validate reference gene stability using multiple candidates (e.g., geNorm or NormFinder analysis) prior to data normalization.

Importantly, the physiological relevance of the concentrations used in this study should be interpreted in the context of the pharmacokinetic and bioavailability profiles of Epi and HT. Following oral administration, Epi is rapidly absorbed and extensively metabolized, with peak plasma concentrations typically reported in the sub-micromolar range (~0.1–1.0 µM), and only transiently reaching higher levels under flavanol-rich dietary conditions [43]. Similarly, HT exhibits rapid absorption and clearance, with circulating levels generally observed in the low nanomolar to sub-micromolar range, predominantly in the form of conjugated metabolites [19,44]. These concentrations are often lower than those employed in in vitro systems, highlighting a limitation in directly extrapolating the present findings to in vivo conditions [45]. Therefore, the experimental concentrations used in this study should be interpreted with caution, and future investigations incorporating physiologically relevant dosing, metabolite-specific activity, and in vivo validation, particularly using ApoE−/− mouse models, will be essential to better assess the translational applicability of these compounds.

Furthermore, it is important to consider that atherosclerosis is a multifactorial process driven by dynamic interactions between macrophages and vascular endothelial cells [46]. While the present study provides insights into macrophage-mediated mechanisms using THP-1 cells, it does not fully capture the complexity of macrophage–endothelium crosstalk, which plays a critical role in the initiation and progression of atherosclerotic lesions. Endothelial dysfunction and activation are key early events that regulate monocyte adhesion, migration, and subsequent foam cell formation [38,47]. Therefore, future studies should evaluate the effects of Epi and HT, individually and in combination, on human endothelial cell models such as human umbilical vein endothelial cells (HUVECs), as well as in co-culture systems that better recapitulate the vascular microenvironment [48,49]. Such approaches would provide a more comprehensive understanding of the impact of these polyphenols on both macrophage and endothelial function and strengthen the translational relevance of the findings.

Another limitation of this study is the exclusive use of THP-1 macrophages, which do not fully recapitulate the complexity of the vascular microenvironment. Functional assays such as Dil-ox-LDL uptake or foam cell formation analysis were not performed, nor did we include endothelial–macrophage co-culture models. However, our mRNA findings for CD36, LOX-1, and ABCA1 are consistent with published functional data demonstrating that hydroxytyrosol reduces ox-LDL uptake and foam cell formation in THP-1 macrophages [41]. Our future studies will incorporate primary human macrophages, endothelial–macrophage co-culture systems, and functional assays (Dil-ox-LDL uptake, Oil Red O staining) to confirm these findings at the functional level. Future investigations will validate these transcriptional findings using functional assays (foam cell formation, cholesterol quantification) and more complex models such as endothelial–macrophage co-culture or animal models of atherosclerosis.

## 4. Materials and Methods

### 4.1. Chemoinformatic Prediction

Chemoinformatics tools helped to predict the suitability of bioactive molecules (Epi and HT) as drugs. SwissADME was used to evaluate the physicochemical descriptors by calculating the ADME features [25]. SwissTarget prediction was used to foresee the protein targets of biomolecules by analyzing a combination of 2D and 3D structural data with electrochemical complementarity [50].

### 4.2. PPI Network Analysis

A protein–protein interaction (PPI) network was constructed to explore the functional association between ICAM-1, VCAM-1, MCP-1 (CCL2), LOX-1 (OLR1), CD36, and GM-CSF (CSF2) using the STRING database (version 12.0) [24]. The analysis was performed by selecting Homo sapiens as the reference organism, with a high confidence interaction score threshold of 0.700. Interaction sources included experimental evidence, curated databases, co-expression, co-occurrence, and text mining data.

### 4.3. Molecular Docking and Potential Binding Site Prediction

The three-dimensional structure was retrieved for all 6 target proteins, namely ICAM-1, VCAM-1, MCP-1, GM-CSF, CD36, and LOX-1, from the RCSB protein data bank (1IAM, 1VSC, 2NZ1, 6BFQ, 5LGD, and 6TLA, respectively). The 3D structure of the chemical ligands of interest was downloaded from the NCBI’s PubChem database (epicatechin-CID: 72276 and hydroxytyrosol-CID: 82755). The proteins were prepared and then docked using AutoDock 4.2 [51]. Each protein structure was analyzed by selecting a chain, removing water molecules, and determining the protein’s binding site grid. The free energy calculations and evaluation of binding configurations of ligand–protein interaction were done. The ideal binding of docking was selected at the lowest energy (kcal/mol) and root mean square deviation (RMSD) [21]. The interactions were further checked using the protein–ligand interaction profiler [52], and the results were tabulated. Structure visualization and figure generation were done using Schrodinger’s PyMOL 2.3.3. [53] and Ligplot+ v.2.2 [54].

### 4.4. THP-1 Monocytic Cell Culture and Differentiation

The human acute monocytic leukemia cell line THP-1 is extensively utilized in research on cardiovascular diseases. THP-1 was obtained from the King Faisal Specialist Hospital and Research Center (KFSH&RC) in Riyadh, Saudi Arabia. THP-1 monocytes were cultured in complete RPMI-1640 tissue culture medium containing 200 mM L-glutamine (1% *v*/*v*), supplemented with fetal bovine serum (FBS, 10% *v*/*v*), penicillin (100 U/mL), and streptomycin (100 μg/mL) (Gibco^TM^, Thermo Fisher Scientific, NY, USA). For subculture, cells were transferred from a flask to a 50 mL Falcon tube and centrifuged at 250× *g* for 5 min at room temperature. The supernatant was then discarded, and the cell pellet was resuspended in 5–7 mL of complete medium and transferred to a small flask. The flask was placed into a 37 °C humidified incubator containing 5% (*v*/*v*) CO_2_. For experimental use, cells between passages 4 and 9 were used.

THP-1 monocytes were differentiated into macrophages by incubating them with 0.16 µL/mL of phorbol 12-myristate 13-acetate (PMA) (Thermo Fisher, GmbH, Germany) for 24 h at 37 °C, 5% (*v*/*v*) CO_2_ [37]. To ensure that differentiation had taken place, the cells were examined under a microscope after 24 h for changes in morphological features that are associated with the process. The culture media supplemented with the PMA was then discarded, and new complete media were added with target treatment (mediators) in the adherent macrophage cells to stop the effect of PMA.

(−)-Epi (≥99% HPLC, HY-N0001; MedChemExpress, NJ, USA) was dissolved in DMSO, and a concentration of 100 mg/mL stock was prepared [55]. HT (≥99.60% HPLC, HY-N0570; MedChemExpress, NJ, USA) was dissolved in DMSO, and a concentration of 125 mg/mL stock was prepared [39]. DMSO therefore served as a control (vehicle). For both mediators, the stock solution was then aliquoted and stored at −20 °C until used. The working solutions were freshly prepared in complete culture medium, and further dilution was done to 0.1% (*v*/*v*) DMSO for all experiments. The incubation of cells treated with Epi alone, HT alone, Epi and HT combined, and control was carried out in a humidified 5% (*v*/*v*) CO_2_ incubator at 37 °C for 24 h.

### 4.5. Cytotoxicity Assays

The lactate dehydrogenase (LDH) assay was used to assess cell viability [56] (cat. no. 88953; Thermo Fisher Scientific, NY, USA). The assay was carried out according to the manufacturer’s instructions. THP-1 macrophages (1 × 10^5^ cells/cm^2^) were seeded into 96-well plates and incubated with mediators or DMSO vehicle control for 24 h at 37 °C and 5% (*v*/*v*) CO_2_ [31,32]. They were treated with Epi and HT diluted in culture media at 5–125 µM concentrations in 0.1% of DMSO to treat the macrophages for 24 h. Following the manufacturer’s instructions, 50 µL of the cell supernatant was transferred to a new 96-well plate, and 50 µL of assay buffer was added and incubated for 30 min at room temperature in the dark. The reaction was then stopped by adding 50 µL of the stop solution and mixed. The cellular LDH concentration was measured using a microplate reader at 490 nm absorbance (BioTek Synergy HT, Agilent Technologies, CA, USA). The results were expressed as a percentage to the DMSO vehicle control, which was arbitrarily assigned as 100%.

### 4.6. Cell Proliferation Assay

Crystal violet (CV) dye was used to react with the DNA of viable cells [56]. The remaining cells from the LDH assay experiment were stained with 0.2% (*w*/*v*) CV solution (in 10% (*v*/*v*) ethanol) and incubated for 5 min at room temperature [32]. The macrophage cells were then washed gently 2–3 times with 100 µL of 1× PBS, and then 50 µL of the solubilization buffer (0.1 M NaH_2_PO_4_ in 50% ethanol solution) was added to dissolve the intracellular CV. The plate was shaken for 5 min before reading absorbance with a microplate reader at 570 nm. The data was shown as a percentage relative to the control, which was designated as 100%.

### 4.7. Monocyte Migration Assay

The migration assay involved comparing the migration of THP-1 monocytic cells in response to the MCP-1 in the presence of a vehicle control or the treatment. Migration was performed using the Boyden chamber assay wherein 8 μm pore filter inserts in 12-well companion plates were used. The cell culture method mimics the artery’s endothelium. THP-1 monocytes at a concentration of 5 × 10^5^ cells were suspended in 1 mL of complete growth medium (RPMI medium 1640) containing either treatment or DMSO and placed on top of the culture insert in the modified Boyden chamber [31]. In the bottom chamber, 1 mL of complete media containing 20 ng/mL of MCP-1/Monocyte Chemotactic and Activating Factor (Sigma-Aldrich, MO, USA; cat. no. SRP3109; 35224: SPL Life Sciences, Pocheon-si, Korea) in every well without the negative control well that had no chemokine. They were immediately incubated at 5% (*v*/*v*) CO_2_ for 3 h to allow the migration of cells. After 3 h of incubation at 37 °C, the remaining cells on the top of the insert membrane were removed. The cells that migrated to the bottom chambers were collected and transferred into tubes and subjected to centrifugation at 250× *g* for 5 min at room temperature. The cell pellet was resuspended in 1 mL of fresh media and then counted by a hemocytometer. Monocyte migration was compared to the number of cells that moved in response to the chemokine alone.

### 4.8. Quantitative Real-Time Polymerase Chain Reaction

Quantitative polymerase chain reaction (qPCR) is used for the analysis of gene expression by measuring the amount of a specific RNA (target). This is achieved by monitoring the amplification of reactions using fluorescence. Three groups of THP-1 macrophages were taken (control, IFN-γ, and IFN-γ treated with mediator for 24 h). Inflammation was induced in all groups without control with 0.13 μL of human interferon γ (IFN-γ, cat. no. 1265; 1 mg/mL, Sigma-Aldrich) for 3 h. Total RNA was extracted from all experimental groups using the Trizol^®^ Plus RNA purification kit (cat. no. 12183555; Thermo Fisher Scientific, USA) according to the manufacturer’s instructions. The isolated RNA was subsequently reverse-transcribed into complementary DNA (cDNA) using Invitrogen SuperScript IV Master Mix (2X) (cat. no. 11756500; Thermo Fisher Scientific, NY, USA) following the manufacturer’s protocol.

qPCR was done by the SYBR Green PCR Master Mix Kit (cat. no. 4309155; Thermo Fisher Scientific, NY, USA) according to the manufacturer’s instructions. A sequence of primers was designed by NCBI BLAST (v 2.17.0) based on exon–exon junctions to prevent amplification of genomic DNA. Amplification of cDNA samples was carried out using the Bio-Rad CFX96 Real-Time PCR Detection System (Bio-Rad Laboratories, Hercules, CA, USA).

Target gene (ICAM-1, MCP-1, and LOX-1) expression was analyzed by a Step One Plus^TM^ real-time PCR system (Applied Biosystems, Waltham, MA, USA). Relative quantification of their expression with fold change and *p*-value was calculated using the comparative threshold method (Ct, 2^−ΔΔCt^) after normalization with the glyceraldehyde-3-phosphate dehydrogenase (GAPDH) housekeeping gene. Table 3 shows lists of primers that were used.

### 4.9. Intracellular Reactive Oxygen Species (ROS) Measurement

To measure ROS production/activity, the 2′,7′-dichlorofluorescin diacetate (DCFDA) cellular ROS detection kit was used (CA1410; Solarbio, Beijing, China). The procedures were carried out according to the manufacturer’s instructions. The assay uses a cell-permeable fluorescent probe 2,7-dichlorodihydrofluorescein diacetate (DCFH-DA), a fluorogenic dye that measures ROS activity within cells [57]. PMA-differentiated THP-1 macrophages (1.5 × 10^5^ cells/cm^2^) were cultured overnight in 96 wells in a microplate at 37 °C and 5% (*v*/*v*) CO_2_. Cells were then incubated in serum-free medium containing 20 μM DCHF-DA at 37 °C in a humidified incubator containing 5% CO_2_ for 45 min in darkness. The cells were then washed with PBS. After this washing step, the cells were treated with the required concentrations of Epi and HT alone and in combination and DMSO as a vehicle control in complete medium. The cells were then incubated for 3 h at 37 °C in a humidified incubator containing 5% CO_2_ in darkness. The fluorescence was then measured at Ex488/Em525 using a microplate reader (Synergy H1; BioTek, CA, USA).

### 4.10. Statistical Analysis

Descriptive analysis as the mean ± standard error of the mean (SEM) of three replicate independent experiments was done. One-way analysis of variance (ANOVA) was performed followed by Tukey’s post hoc test to compare the differences among means using the analytic tools of GraphPad Prism version 8.0.1 (GraphPad Software Inc., San Diego, CA, USA). The significance was determined based on *p*-values: ns (non-significant), * *p* ≤ 0.01 (significant), ** *p* ≤ 0.001 (more significant), and *** *p* ≤ 0.0001 (highly significant).

## 5. Conclusions

The anti-inflammatory, antioxidant, and anti-atherogenic effects of Epi and HT in combination could potentially be used for the treatment of atherosclerotic diseases. They may be used as a nutritional supplement for treating inflammatory diseases by reducing damage from chronic inflammation and modulating important biomarker genes in the early stages of atherosclerosis. Notably, our work demonstrates that combined treatment exerts a more pronounced effect than individual compounds, significantly inhibiting monocyte migration and reducing ROS production. These findings provide evidence of a coordinated enhanced effect and offer new insight into their potential role in modulating early atherogenic events. Future studies incorporating endothelial cell models and physiologically relevant dosing conditions are warranted to validate these findings. Further in vivo investigations (e.g., using ApoE−/− mouse models) to validate their therapeutic efficacy will contribute to improved comprehension of the molecular processes behind the anti-inflammatory properties of natural substances and their application as dietary supplements. Future studies are needed to fully evaluate their efficacy in long-term feeding studies using atherosclerotic mouse models to examine effects on adipose tissue, plaque size, immune cell proliferation in bone marrow, and gene expression. Cytotoxicity of the phytocompounds will be checked on other human cell lines too.

## Figures and Tables

**Table 1:** Physiochemical properties of Epi and HT based on SwissADME tool.

Physicochemical Properties	Epicatechin (Epi)	Hydroxytyrosol (HT)
MLogP	0.24	0.60
Molecular weight (g/mol)	290.27	156.16
Number of H-bond acceptors	6	3
Number of H-bond donors	5	3
Number of rotatable bonds	1	2

Note: LogP refers to the octanol–water partition coefficient.

**Table 2:** Docking results of ligand compounds (Epi and HT) with selected inflammatory proteins.

Protein	PDB	Ligand	Binding Energy (kcal/mol)	Inhibition Constant	Residue Interaction (Å)	
					H-Bonds	Others
ICAM-1	1IAM	Epi	−4.69	365.94 μM	Thr85N-O2 (2.83 Å), Oγ1-O2 (2.73 Å), Oγ1-O3 (3.11 Å)	Pro12, Arg13, Gln58, Tyr83, Trp84
		HT	−4.76	323.52 μM	Glu34O-O1 (2.43 Å), Ser61Oγ-O2 (2.82 Å), Oγ-O3 (2.76 Å)	Thr35, Pro 36, Gln62, Pro63, Met64
VCAM-1	1VSC	Epi	−3.64	2.14 mM	Val47N-O2 (2.94 Å), Ser411OG-O6 (3.21 Å)	Trp35, Ser41, Pro42, Leu43, Gly45, Lys46, Glu96, His98, Lys112
		HT	−5.21	151.80 μM	Pro42O-O1 (2.85 Å), His67 NE2-O1 (2.67 Å), Thr151OG1-O2 (2.67 Å)	Ser41, Asn44, Gly64
MCP-1	AF-P-13500-F1	Epi	−5.65	72.53 μM	Tyr36Nam-O3 (3.68 Å), Asn37Nam-O3 (3.83 Å), Thr39Nam-O3 (3.09 Å), Thr39O3-O3 (2.95 Å), Asn40Nam-O3 (3.23 Å), Glu73O-O2 (2.81 Å), Cys75Nam-O3 (3.55 Å)	Ile74, Asn37
		HT	−4.83	285.95 μM	Ser50O-O3 (3.80 Å), Tyr51O-O3 (2.71 Å), Arg52Ng^+^-O3 (3.77 Å), Glu73O-O3 (2.71 Å), Glu73O-O3 (2.49 Å)	Lys67, Arg52, Ile65
GM-CSF	6BFQ	Epi	−4.39	600.87 μM	Asn37O-O6 (2.54 Å), Tyr59N-O2 (3.09 Å), Glu104N-O5 (2.80 Å)	Met36, Phe85, Thr102, Phe103
		HT	−4.71	353.74 μM	AlaN-O1 (2.95 Å)	Arg32, Arg33, Leu34, Ala53, Glu41, Tyr50, Ser92, Gly97, Ile100
CD36	5LGD	Epi	−5.22	149.45 μM	Asn159ND2-O2 (2.88 Å), Met156O-O3 (2.96 Å), Glu397OE1-O5 (2.86 Å), Lys398O-O2 (2.94 Å)	Lys403, Ile673, Asp676, Thr677
		HT	−5.04	202.61 μM	Lys398O-O2 (2.63 Å), Glu672OE2-O1 (2.88 Å)	Met156, Asn159, Ser160, Leu669, Ile673, Asp6
LOX-1	6TLA	Epi	−6.93	8.35 μM	Ser198O-O5 (2.65 Å), Arg248NH1-O4 (3.25 Å)	Ile195, Ser196, Ser199, Phe200, Ser212, Tyr213, Glu247, Gly249
		HT	−5.84	52.54 μM	Glu192OE1-O1 (2.58 Å), Glu192O-O1 (2.99 Å), Ser198O-O3 (2.63 Å)	Ile195, Ser196, Tyr213, Gln247, Arg48, Gly249

**Table 3:** Primer sequence used in qPCR for human genes.

Gene	Forward Primer (5′-3′)	Reverse Primer (5′-3′)
ICAM-1	GACCAGAGGTTGAACCCCAC	GCGCCGGAAAGCTGTAGAT
MCP-1	TCTCGCCTCCAGCATGAAAG	GGCATTGATTGCATCTGGCT
LOX-1	TGCTTCACTCTCTCATTCTTAGCTT	GGCACCACCATGGAGAGTAA
GAPDH	CTTTTGCGTCGCCAGCCGAG	GCCCAATACGACCAAATCCGTTGACT

## Data Availability

The original contributions presented in this study are included in the article. Further inquiries can be directed to the corresponding author.

## Supplementary Materials

The following supporting information can be downloaded at: https://www.mdpi.com/article/10.3390/ijms27104235/s1.

## Abbreviations

The following abbreviations are used in this manuscript:

CD36	cluster of differentiation antigen-36
CV	crystal violet
CVD	cardiovascular disease
Epi	epicatechin
GAPDH	glyceraldehyde-3-phosphate dehydrogenase
GM-CSF	granulocyte–macrophage colony-stimulating factor
HT	hydroxytyrosol
ICAM-1	intercellular adhesion molecule-1
Ki	inhibition constant
LDH	lactate dehydrogenase
LDL	low-density lipoprotein
LOX-1	lipoprotein receptor-1
MCP-1	monocyte chemoattractant protein-1
NADPH	nicotinamide adenine dinucleotide phosphate
NF-κB	nuclear factor kappa-light chain enhancer of activated B cells
ox-LDL	oxidized LDL
PPI	protein–protein interaction
PMA	phorbol 12-myristate 13-acetate
qPCR	quantitative polymerase chain reaction
ROS	reactive oxygen species
SR-AI	scavenger receptor class AI
VCAM-1	vascular cell adhesion molecule-1
DCFH-DA	2,7-dichlorodihydrofluorescein diacetate
SEM	standard error of the mean
IFN-γ	interferon gamma
DMSO	dimethyl sulfoxide
H_2_O_2_	hydrogen peroxide
