# Supplementary material for: Enhanced Anti-Atherogenic Effects of Epicatechin and Hydroxytyrosol in THP-1 Macrophages: An Integrated In Silico and In Vitro Study
Source: Int J Mol Sci. 2026 May 10;27(10):4235. doi: 10.3390/ijms27104235 (PMC13207789; doi:10.3390/ijms27104235)
Supplement: Supplementary file 1 [file ijms-27-04235-s001.zip › ijms-4273652-supplementary.pdf]

Table S1: The raw Ct values for GAPDH. GAPDH expression was stable across all experimental groups in THP-1 cells treated with epicatechin and hydroxytyrosol.

**Experimental Group**	**Mean GAPDH Ct**	**Standard Deviation (SD)**
Control	19.17	0.01
IFN-γ	20.84	0.02
IFN-γ + HT	19.47	0.07
IFN-γ + Epi	19.78	0.03
IFN-γ + Epi + HT	19.65	0.09

The maximum variation in mean Ct across all groups was less than **0.5 cycles**, and all individual SD values were ≤0.25.
